# Bioactive Natural Spirolactone-Type 6,7-*seco*-*ent*-Kaurane Diterpenoids and Synthetic Derivatives

**DOI:** 10.3390/molecules23112914

**Published:** 2018-11-08

**Authors:** Haonan Li, Runwei Jiao, Jiahui Mu, Shengtao Xu, Xu Li, Xianhua Wang, Zhanlin Li, Jinyi Xu, Huiming Hua, Dahong Li

**Affiliations:** 1 Key Laboratory of Structure-Based Drug Design & Discovery, Ministry of Education, and School of Traditional Chinese Materia Medica, Shenyang Pharmaceutical University, Shenyang 110016, China; lihaonan073@163.com (H.L.); jiaorunwei@163.com (R.J.); 13130821899@sina.cn (J.M.); lzl1030@hotmail.com (Z.L.); huimhua@163.com (H.H.); 2 Department of Medicinal Chemistry and State Key Laboratory of Natural Medicines, China Pharmaceutical University, Nanjing 210009, China; cpuxst@163.com (S.X.); jinyixu@china.com (J.X.); 3 Jiangsu Kanion Pharmaceutical Co., Ltd., Lianyungang 222001, China; lixu19900611@163.com; 4 School of Public Health, Qingdao University, Qingdao 266021, China; xianhuawang126@sina.com

**Keywords:** spirolactone-type, 6,7-*seco*-*ent*-kaurane, diterpenoid, natural product, synthetic derivative

## Abstract

Diterpenoids are widely distributed natural products and have caused considerable interest because of their unique skeletons and antibacterial and antitumor activities and so on. In light of recent discoveries, *ent*-kaurane diterpenoids, which exhibit a wide variety of biological activities, such as anticancer and anti-inflammatory activities, pose enormous potential to serve as a promising candidate for drug development. Among them, spirolactone-type 6,7-*seco*-*ent*-kaurane diterpenoids, with interesting molecular skeleton, complex oxidation patterns, and bond formation, exhibit attractive activities. Furthermore, spirolactone-type diterpenoids have many modifiable sites, which allows for linking to various substituents, suitable for further medicinal study. Hence, some structurally modified derivatives with improved cytotoxicity activities are also achieved. In this review, natural bioactive spirolactone-type diterpenoids and their synthetic derivatives were summarized.

## 1. Introduction

Natural products have been used to treat various diseases in China for hundreds of years. Their novel molecular skeletons and promising cytotoxic activities are invaluable sources for drug discovery and development processes [1,2,3,4]. Natural products have played crucial roles in drug discovery. Besides natural products, natural product-derived compounds are also important to cancer therapy [5,6,7,8,9,10,11]. It is well known that diterpenoids are structurally diverse and widely distributed natural compounds, they have attracted interest from the scientific community because of their unique skeletons and therapeutic effects—antitumor [12,13], anti-inflammatory [14], antibacterial [15,16], and so on [17,18,19,20,21]—especially for anticancer agents, such as the most famous anticancer natural compound paclitaxel [22,23,24,25].

**Figure 1:**
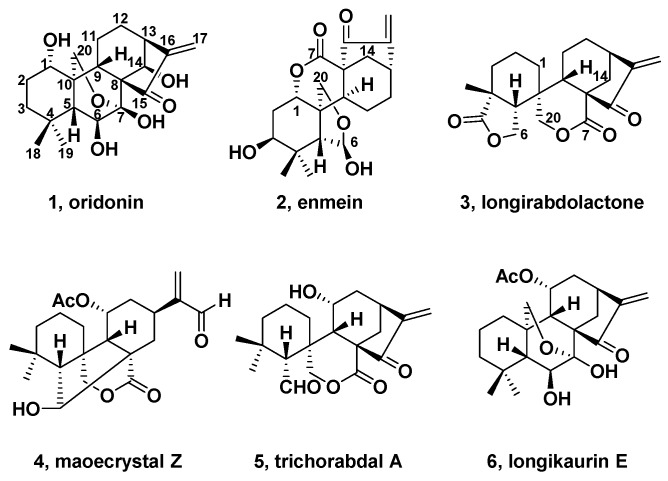
Structures of oridonin (**1**), enmein (**2**), longirabdolactone (**3**), maoecrystal Z (**4**), trichorabdal A (**5**), and longikaurin E (**6**).

*ent*-Kaurenes, such as oridonin (**1**, Figure 1), have been investigated for more than 40 years [26,27,28,29]. Moreover, in 2015, the *ent*-kaurane diterpenoid derivative HAO472, l-alanine-(14-oridonin) ester trifluoroacetate, was advanced to Phase I human clinical trial in China to cure acute myelogenous leukemia [30]. There are two subtypes of 6,7-*seco*-*ent*-kauranes, spirolactone-type (**7**,**20**-lactone) and enmein-type (**1**,**7**-lactone) [31,32]. Particularly, spirolactone-type diterpenoids have distinct chemical skeletons and demonstrate important bioactivities which have attracted great interest from experts and scholars. Before the 1980s, spirolactone-type diterpenoids were misidentified as enmein-type diterpenoids. Until the mid-1980s, misidentifications were corrected with the development of modern 2D NMR spectra [33,34]. The first isolation of spirolactone-type diterpenoids was in 1981 [35]. After that, in order to isolate antitumor diterpenoids, more and more spirolactone-type diterpenoids were isolated from Isodon plants of the Labiatae family [36,37,38,39,40,41,42,43,44,45,46,47,48,49,50,51,52,53,54,55], but natural sources were limited.

The first total synthesis of 6,7-*seco*-*ent*-kauranoid enmein (**2**, Figure 1) was achieved by Fujita et al. which was a landmark [56,57]. After that, a semisynthesis of longirabdolactone (**3**, Figure 1) was achieved in 2003 [58]; maoecrystal Z (**4**, Figure 1), trichorabdal A (**5**, Figure 1), and longikaurin E (**6**, Figure 1) were achieved in 2011 to 2014 [59,60]. By the end of September 2018, three reviews summarized the total synthesis of Isodon diterpenes [61,62,63].

The Sun [64] and Pu Group [65] had reviewed “diterpenoids from the Isodon genus”. They comprehensively summarized isolation, structural elucidation, and total synthesis of spirolactone-type diterpenoids. In this review, bioactive spirolactone-type natural products and the synthetic medicinal chemistry work will be summarized.

## 2. Natural Bioactive Spirolactone-Type Diterpenoids

By the end of October 2018, 105 spirolactone-type diterpenoids have been isolated from Isodon species. Several exhibited biological activities and are summarized below.

**Figure 2:**
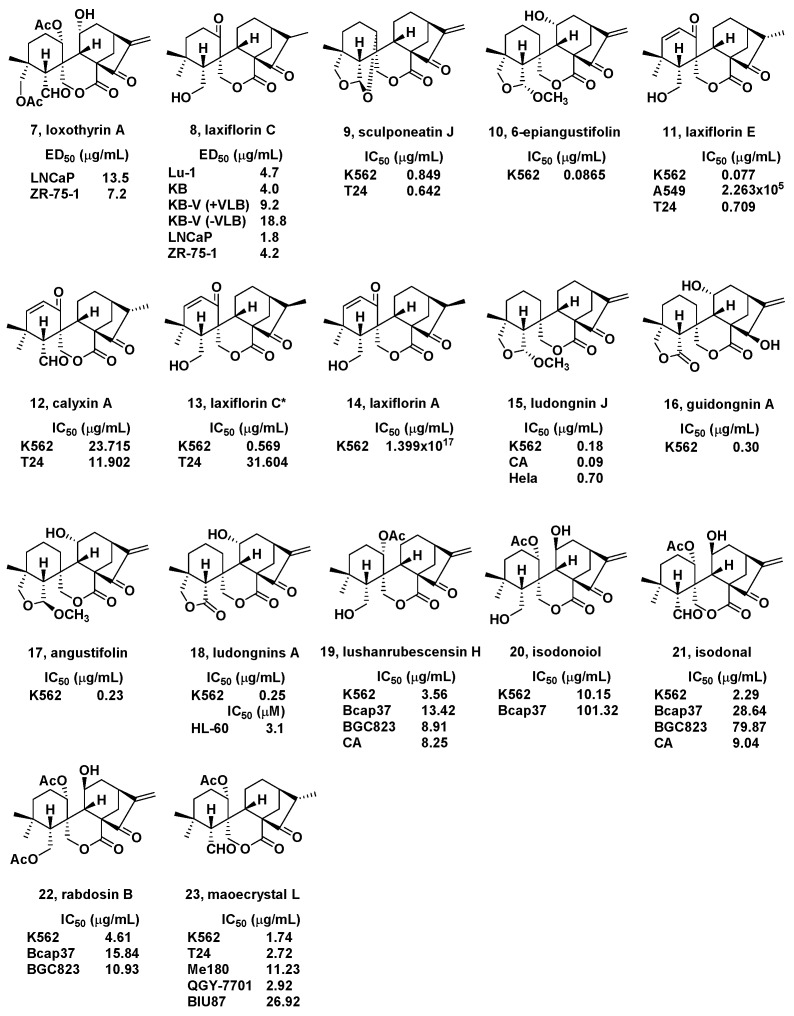
Bioactive natural spirolactone-type diterpenoids **7**–**23**.

In 1995, loxothyrin A (**7**, Figure 2) was isolated by Sun’s Group from the leaves of *I. loxothyrsa* [66]. It showed cytotoxicity effects toward hormone-dependent human prostatic LNCaP and breast ZR-75-1 cancer cell lines with ED_50_ values of 13.5 and 7.2 μg/mL, respectively.

In the same year, laxiflorins A–C (**8**, Figure 2) were isolated from *I. eriocalyx* var. *laxiflora.* by Sun and coworkers [67]. Cytotoxic activities were shown against human lung cancer Lu-1, human oral epidermoid carcinoma KB, vinblastine-resistant KB KB-V, LNCaP, and ZR-75-1 cells. They showed cytotoxicity with ED_50_ values from 1.8 to 18.8 μg/mL.

Two new spirolactone-type diterpenoids were isolated from *I. sculponeatus* by Jiang and coworkers in 2002 [68]. All diterpenoids were tested against K562 (chronic myelogenous leukemia) and T24 (bladder cancer) cells. Among them, sculponeatin J (**9**, Figure 2) showed inhibitory effects (IC_50_) of 0.849 and 0.642 μg/mL against K562 and T24 cells, respectively.

Four new *ent*-kaurane diterpenoids were isolated from the *I. enanderianus* in the same year by and coworkers [69]. Among which, a new spirolactone-type diterpenoid was named 6-epiangustifolin (**10**, Figure 2), and tested for its cytotoxicity toward K562 cells. The results showed that **10** exhibited inhibitory activity with an IC_50_ value of 0.0865 μg/mL against the K562 cell line, which was stronger than *cis*-platin, the positive reference.

One new spirolactone-type diterpenoids, laxiflorin E, and four known ones were isolated from *I. eriocalyx* var. *laxiflora*. by Niu et al. in 2002 [70]. All isolates were tested for antiproliferative activities toward K562, lung cancer A549, and T24 human cancer cell lines. Among them, laxiflorin E, calyxin A, laxiflorin C*, and laxiflorin A (**11**–**14**, Figure 2) displayed cytotoxic activity with IC_50_ values from 0.077 to 1.399 × 10^17^ μg/mL.

Han et al. isolated five new and eight known spirolactone-type diterpenoids from *I. rubescens* var. *lushiensis* in 2003. The cytotoxicity of most isolates were tested against K562 cell line. Among which, ludongnin J, guidongnin A, angustifolin, and ludongnin A (**15**–**18**, Figure 2) showed significant inhibitory effects with IC_50_ values from 0.18 to 0.83 μg/mL. Furthermore, compound **15** also exhibited inhibitory activities against liver cancer CA and uterine cervix cancer Hela cell lines with IC_50_ values below 0.70 μg/mL [71]. Moreover, in 2010, Luo et al. also found that compound **18** exhibited cytotoxicity against promyelocytic leukemia HL-60 cells with an IC_50_ value of 3.1 μM [72].

In the same year, Han et al. also isolated two new and four known spirolactone-type diterpenoids from *I. rubescens* var. *lushiensis* [73]. All isolates were tested for their cytotoxic effects against K562, human breast cancer Bcap37, CA, human nasopharyngeal cancer CNE, human cystic cancer BIU87, human stomach cancer BGC823, and Hela cell lines. Lushanrubescensin H, isodonoiol, isodonal, and rabdosin B (**19**–**22**, Figure 2) displayed cytotoxic activities with IC_50_ values from 2.29 to 28.64 μg/mL.

Three new, together with six known, spirolactone-type diterpenoids were isolated by Shen and coworkers from *I. eriocalyx* (Dunn.) Hara in 2005 [74]. The cytotoxicity against T-24, K562, Me180 (human cervical epithelial cancer), QGY-7701 (human hepatoma), and BIU87 cell lines. Among them, maoecrystal L (**23**, Figure 2) showed strong cytotoxicity with IC_50_ values of 2.72, 1.74, 11.23, 2.92, and 26.92 μg/mL, respectively.

**Figure 3:**
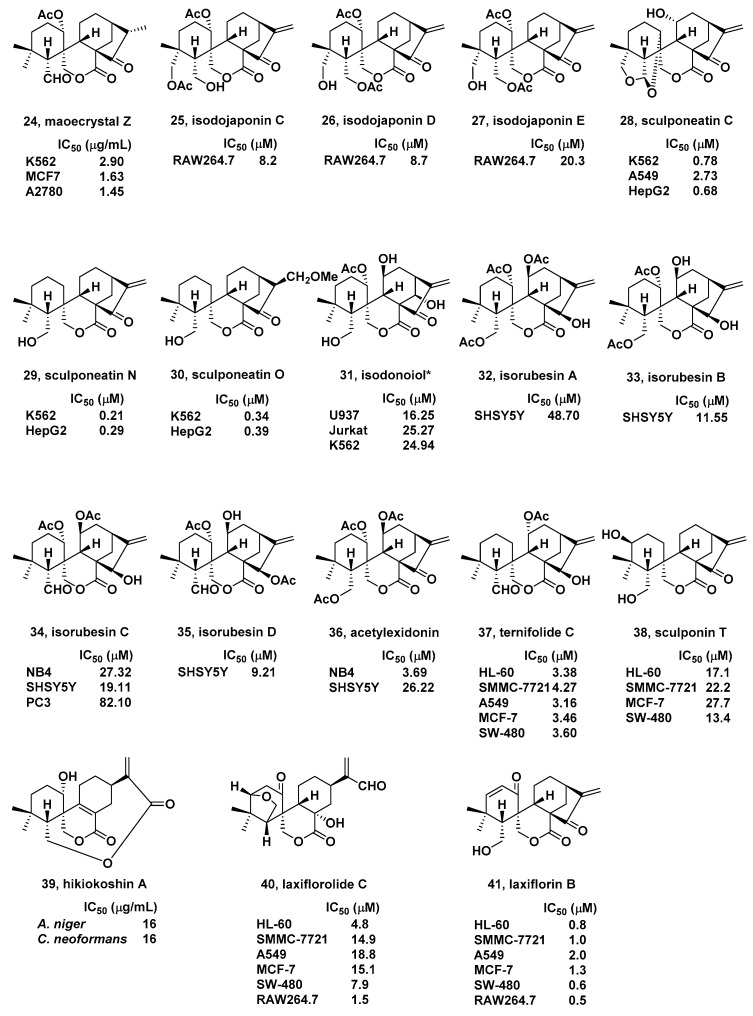
Chemical structures of natural spirolactone-type diterpenoids **24**–**41.**

In 2006, Han and coworkers isolated a novel spirolactone-type diterpenoid, maoecrystal Z (**24**, Figure 3), with an unprecedented skeleton from *I. eriocalyx* (Labiatae) [75]. Fortunately, **24** exhibited comparable inhibitory activities against K562, human breast cancer MCF-7 and human ovarian cancer A2780 cells with IC_50_ values from 1.45 to 2.90 μg/mL.

Three novel spirolactone-type diterpenoids, isodojaponin C–E (**25**–**27**, Figure 3), were isolated by Hong et al. from the aerial parts of *I. japonicus* in 2008 [76]. The inhibitory effects of LPS-induced nitric oxide (NO) production by the isolates were tested in murine macrophage RAW264.7 cells. IC_50_ values were 8.2, 8.7, and 20.3 μM, respectively.

In 2009, a known spirolactone-type diterpenoids, named sculponeatin C (**28**, Figure 3), was isolated from *I. sculponeatus* by Li and coworkers [77]. The results of cytotoxicity test showed that **28** exhibited strong inhibitory activities towards K562, A549, and HepG2 (human hepatoma) cells, with IC_50_ values of 0.78, 2.73, and 0.68 μM, respectively.

Four new spirolactone-type diterpenoids were identified by Li et al. from the aerial parts of *I. sculponeatus* in the 2010 [78]. Among which, sculponeatin N and sculponeatin O (**29** and **30**, Figure 3) displayed strong inhibitory activities (IC_50_) on K562 and HepG2 cell lines between 0.21 and 0.39 μM.

In the same year, Zhang and coworkers isolated one known spirolactone-type diterpenoid, isodonoiol* (**31**, Figure 3), from *I. rubescens* var. *lushanensis* [79]. Interestingly, in cytotoxicity assays, isodonoiol showed moderate inhibitory activities with IC_50_ values above 16.25 μM towards U937 (human histiocytic lymphoma), Jurkat, and K562 cell lines.

Four new spirolactone-type diterpenoids, together with four known ones, were got by Gao’s group from *I. rubescens* in 2011 [80]. The antitumor activities were screened against acute promyelocytic leukemia NB4, A549, neuroblastoma SHSY5Y, prostate cancer PC3, and MCF-7 cells. Among them, isorubesins A–D and acetylexidonin (**32**–**36**, Figure 3) exhibited moderate inhibitory activities (IC_50_ values form 3.69 to 82.10 μM).

In 2012, Zou et al. isolated a new and three known spirolactone-type diterpenoids from *I. ternifolius* [81]. Among them, ternifolide C (**37**, Figure 3) exhibited IC_50_ values of 4.27, 3.38, 3.46, 3.16, and 3.60 μM against hepatocellular carcinoma SMMC-7721, HL-60, MCF-7, A-549, and colon cancer SW-480 cells, respectively.

In 2014 Jiang et al. isolated one new spirolactone-type diterpenoid named sculponin T (**38**, Figure 3) from *I. sculponeatus* [82]. Fortunately, compound **38** showed moderate cytotoxic activities towards human tumor SMMC-7721, HL-60, SW-480, and MCF-7 cells with IC_50_s above 13.4 μM.

Three new spirolactone-type diterpenoids were isolated by Tanaka and coworkers from *I. japonicus* in the same year [83]. Their antifungal activities were evaluated. Particularly, hikiokoshin A (**39**, Figure 3) displayed antifungal activities against *Cryptococcus neoformans* and *Aspergillus niger* with IC_50_ values of 16 μg/mL each.

In 2014, eighteen new spirolactone-type diterpenoids were isolated and determined by Wang and coworkers from *I. eriocalyx* var. *laxiflora* [84]. The cytotoxic effects of all isolates were tested against A-549, SMMC-7721, MCF-7, HL-60, and SW-480 cells. Laxiflorolide C and laxiflorin B (**40** and **41**, Figure 3) exhibited selective cytotoxic activities with IC_50_s between 0.6 and 18.8 μM. Moreover, laxiflorolide C and laxiflorin B also showed inhibitory effects on LPS stimulated NO production in RAW264.7 cells, with IC_50_s of 1.5 and 0.5 μM, respectively.

## 3. Synthetic Spirolactone-Type Diterpenoid Derivatives

**Scheme 1:**
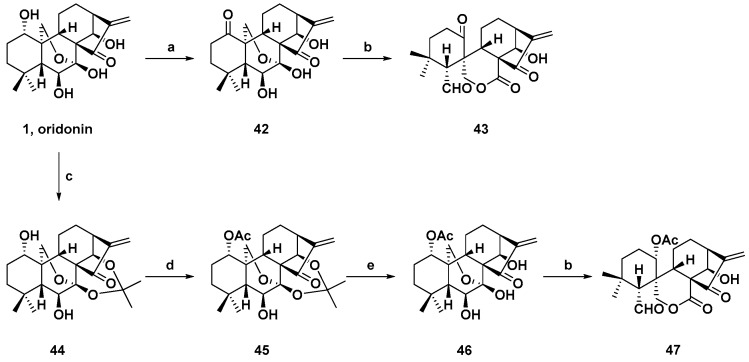
Synthetic route of spirolactone-type diterpenoid skeletons from oridonin. Reagents and conditions: (**a**) Jones reagent, acetone, 0 °C; (**b**) Pb(OAc)_4_, Na_2_CO_3_, THF, rt; (**c**) 2,2-Dimethoxypropane, acetone, TsOH, 56 °C; (**d**) Ac_2_O, TEA, DMAP, rt; and (**e**) 10% HCl, THF, rt.

Though spirolactone-type diterpenoids exhibited cytotoxic effects with interesting molecular skeletons, the amount of spirolactone-type diterpenoids extracted from natural sources could not meet the needs of drug development. In order to achieve large scale compound supply, convenient methods have been built up. Lead tetraacetate was used as oxidation to finish C-6 and C-7 bond cleavage of commercially available oridonin to produce spirolactone-type core structure. The synthesis routine is illustrated in Scheme 1. Based on this core, diverse spirolactone-type derived compounds could be obtained [85].

**Scheme 2:**
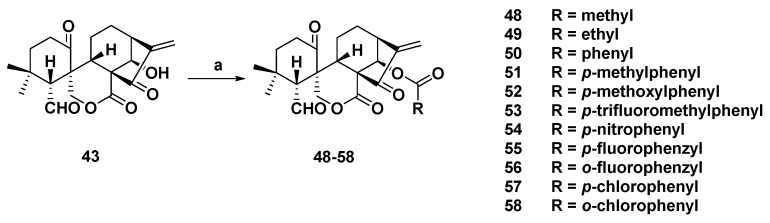
Synthetic route of spirolactone-type diterpenoid analogs (**48**–**58**). Reagents and conditions: (**a**) RCOOH, DMAP, EDCI, rt.

In this way, Wang et al. designed and synthesized a series of novel 14-*O*-derivatives of **43** (Scheme 2). All derivatives were evaluated for their antiproliferative activities against K562, human gastric cancer MGC-803, human esophageal cancer CaEs-17, and human hepatoma Bel-7402 cell lines. The results showed that they exhibited stronger cytotoxicity than **43**. Among them, **51** (Table 1) exhibited the strongest cytotoxicity with IC_50_ values of 1.27, 2.24, 1.05, and 1.54 µM, respectively.

**Scheme 3:**
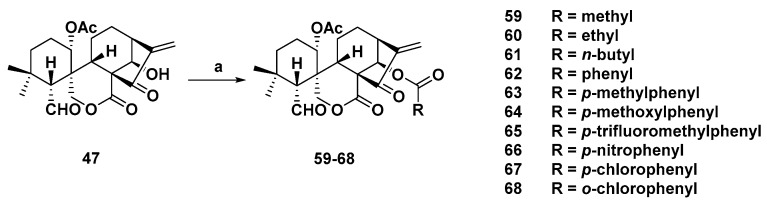
Synthetic route of spirolactone-type diterpenoid analogs (**59**–**68**). Reagents and conditions: (**a**) RCOOH, DMAP, EDCI, rt.

Li and coworkers linked several acids to spirolactone-type core structure with ether bond (Scheme 3) [86]. The antiproliferative activities were tested against the above four cancer cell lines. Target derivatives were also more potent than parent compound oridonin **68** (Table 1) showed the most potent inhibitory activities with IC_50_s below 1.39 µM. The structure–activity relationships (SARs) were also disclosed. When R were alkyl groups (**59**–**61**), with the increased length of R groups, stronger cytotoxicity could be observed in MGC-803 cell line. Furthermore, when R were aromatic groups (**62**–**68**), their activities were stronger than those of alkyl groups, particularly, when substituted by chloro. The most potent **68** was selected to explore antiproliferative mechanism. The results indicated that **68** could induce apoptosis in a dose-dependent fashion and arrest cell accumulation at G2/M phase in Bel-7402 cells.

**Scheme 4:**
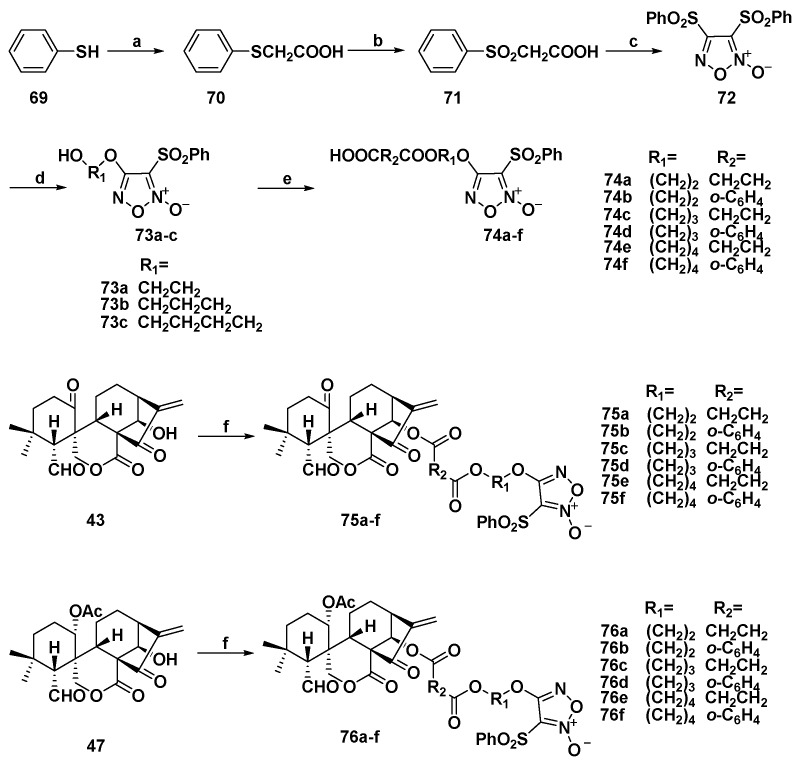
Synthesis of NO donor/spirolactone-type diterpenoid hybrids **75a**–**f** and **76a**–**f**. Reagents and conditions: (**a**) ClCH_2_COOH, NaOH (aq), 60 °C; (**b**) 30% H_2_O_2_, AcOH, rt; (**c**) fuming HNO_3_, AcOH, 60 °C; (**d**) corresponding diol, NaOH (aq), THF, rt; (**e**) triethylamine, succinic anhydride, DMAP, rt; and (**f**) **74a**–**i**, EDCI, DMAP, rt.

In 2016, Xu’s Group synthesized several furozan-based NO-donating derivatives (Scheme 4) [87]. Compared with parent compounds **43** and **47**, all the synthetic target molecules showed improved antiproliferative activities, especially toward Bel-7402 cell line. The SARs revealed when R_2_ was aromatic linkers (**75b**, **75d**, **75f**, **76b**, **76d**, and **76f**), the antiproliferative effects were better than those of alkyl linkers (**75a**, **75c**, **75e**, **76a**, **76c**, and **76e**). Particularly, compound **76d** (Table 1) showed the most potent IC_50_s between 0.86 and 3.75 µM against MGC-803, K562, Bel-7402, and CaEs-17 cells. The NO-releasing properties were evaluated by Griess assay. The results showed that all derivatives more than 15 µM released NO in 1 h which would contribute to their antiproliferative activities. Furthermore, a further mechanism of **76d** was studied in Bel-7402 cells. They found that **76d** could induce cell cycle arrest at the S phase. It was also found that **76d** could decline the mitochondrial membrane potentials which indicated that **76d** induced apoptosis through intrinsic pathways.

**Scheme 5:**
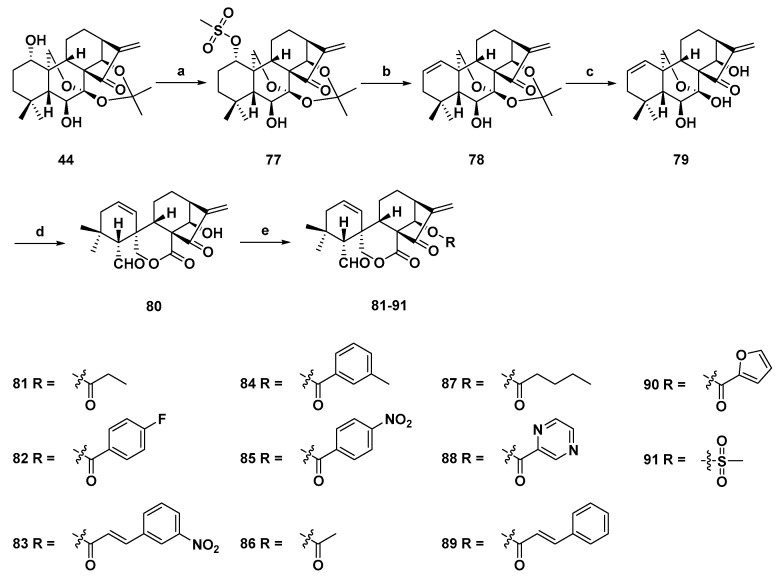
Synthesis of spirolactone-type 6,7-*seco*-*ent*-kauranoid derivatives (**80**–**91**). Reagents and conditions: (**a**) MsCl, TEA, 0 °C; (**b**) LiBr, Li_2_CO_3_, DMF, 110 °C; (**c**) 10% HCl, THF (*v*/*v*, 1:1), rt; (**d**) Pb(OAc)_4_, K_2_CO_3_, THF, 0 °C; and (**e**) EDCI, DMAP, DCM, rt.

**Scheme 6:**
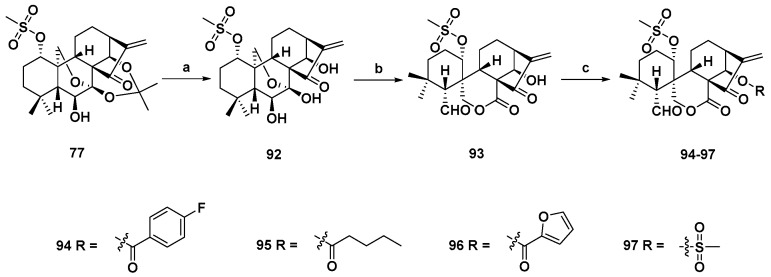
Synthesis of spirolactone-type 6,7-*seco*-*ent*-kauranoid derivatives (**94**–**97**). Reagents and conditions: (**a**) 10% HCl, THF (*v*/*v*, 1:1), rt; (**b**) Pb(OAc)_4_, K_2_CO_3_, THF, 0 °C; and (**c**) EDCI, DMAP, DCM, rt.

In order to discover more bioactive spirolactone-type diterpenoid derivatives, two series of novel derivatives with various substituents at 14-OH were designed and synthesized by Xu et al. in 2017 (Scheme 5 and Scheme 6). The antiproliferative activities of all derivatives were evaluated against four human cancer cell lines (MGC-803, MCF-7, Bel-7402, and K562). Compound **82** (Table 1) exhibited IC_50_s between 0.68 and 2.2 µM, which was the strongest derivatives of this series [88]. The mechanism of action of **82** was also investigated. After treatment with **82**, the mitochondrial membrane potential in MCF-7 cell declined. Western blot results showed that **82** could increase the levels of p-ERK, Bax and caspase 3, and reduced the expression of P53 and Bcl-2. **82** also induced cell accumulated at the G2/M phase. In short, these results illustrate that derivative **82** induced apoptosis through a mitochondria-related pathway.

## 4. Conclusions

In summary, natural spirolactone-type diterpenoids exhibited cytotoxic effects. Its synthetic derivatives showed more potent antiproliferative effects than the corresponding parent compounds. Hence, spirolactone-type diterpenoids are worthy of further research. However, there are few in-depth pharmacological reports on spirolactone-type diterpenoids so far. We hold the view that, for drug exploration, further studies should firstly focus on the detailed mechanism study. Based on these, spirolactone-type diterpenoid derivatives with clear target should be explored. We hope this review can provide useful information in the field of bioactive natural and synthetic spirolactone-type diterpenoids.

## Figures and Tables

**Table 1:** The most potent spirolactone-type diterpenoid derivatives of each series.

Compound	IC_50_ [µM]				
	K562	MGC-803	CaEs-17	Bel-7402	MCF-7
51	1.27	2.24	1.05	1.54	/ ^a^
68	0.39	1.28	0.60	1.39	/ ^a^
76d	1.74	1.16	3.75	0.86	/ ^a^
82	0.69	2.20	/ ^a^	1.80	0.68

^a^ “/” represents not tested.
